# Paraneoplastic Dermatomyositis Revealing the Metastatic Progression of an Undifferentiated Nasopharyngeal Carcinoma: A Case Report From Northern Morocco

**DOI:** 10.7759/cureus.57172

**Published:** 2024-03-29

**Authors:** Hamza Zerbani, Nabila Sellal, Mariame Harrak, Hajar El Bakouri, Sami Amraoui, Mohamed El Hfid

**Affiliations:** 1 Radiation Therapy Department, University Hospital of Tangier, Tangier, MAR

**Keywords:** concomitant chemoradiation therapy, corticosteroid therapy, metastatic progression, paraneoplastic dermatomyositis, nasopharyngeal carcinoma

## Abstract

Dermatomyositis (DM) is an inflammatory disease of striated muscles and skin that can occur sporadically or rarely be associated with malignancy, thereby serving as a potential clinical indicator or harbinger of underlying cancer. Knowing the pathognomonic, clinical, and biological features of DM plays a pivotal role in its recognition. Its correlation with nasopharyngeal carcinoma (NPC) is particularly prevalent in regions where the incidence of NPC is notably high, underscoring the intricate interplay between immune dysregulation and oncogenesis.

Specially, in the context of patients previously treated for NPC, the emergence of DM raises the clinical suspicion of metastatic progression or recurrence of the cancer. Thus, early recognition of DM-associated paraneoplastic syndromes can facilitate prompt intervention and optimize patient outcomes. We present a case of metastatic progression in a patient treated for NPC, revealed by the pathognomonic, clinical, and biological signs of DM.

## Introduction

Dermatomyositis (DM) is a rare myopathy that involves the perivascular tissue of striated muscles [1]. Its etiology remains unknown, often auto-immune, but it can be observed during a neoplastic disease [2]. The first association of DM with cancer was described in 1916 in a patient with gastric adenocarcinoma [2], and since then, it has been reported in several cases of pulmonary, breast, and ovarian tumors, either at the diagnosis or during the course of the disease [3]. Regarding nasopharyngeal tumors, in nations with high incidence, this association is not exceptional, with an estimated one case per thousand cases [4]. We describe a case of metastatic spread of an undifferentiated nasopharyngeal carcinoma (NPC) revealed by paraneoplastic DM.

## Case presentation

**Figure 1:**
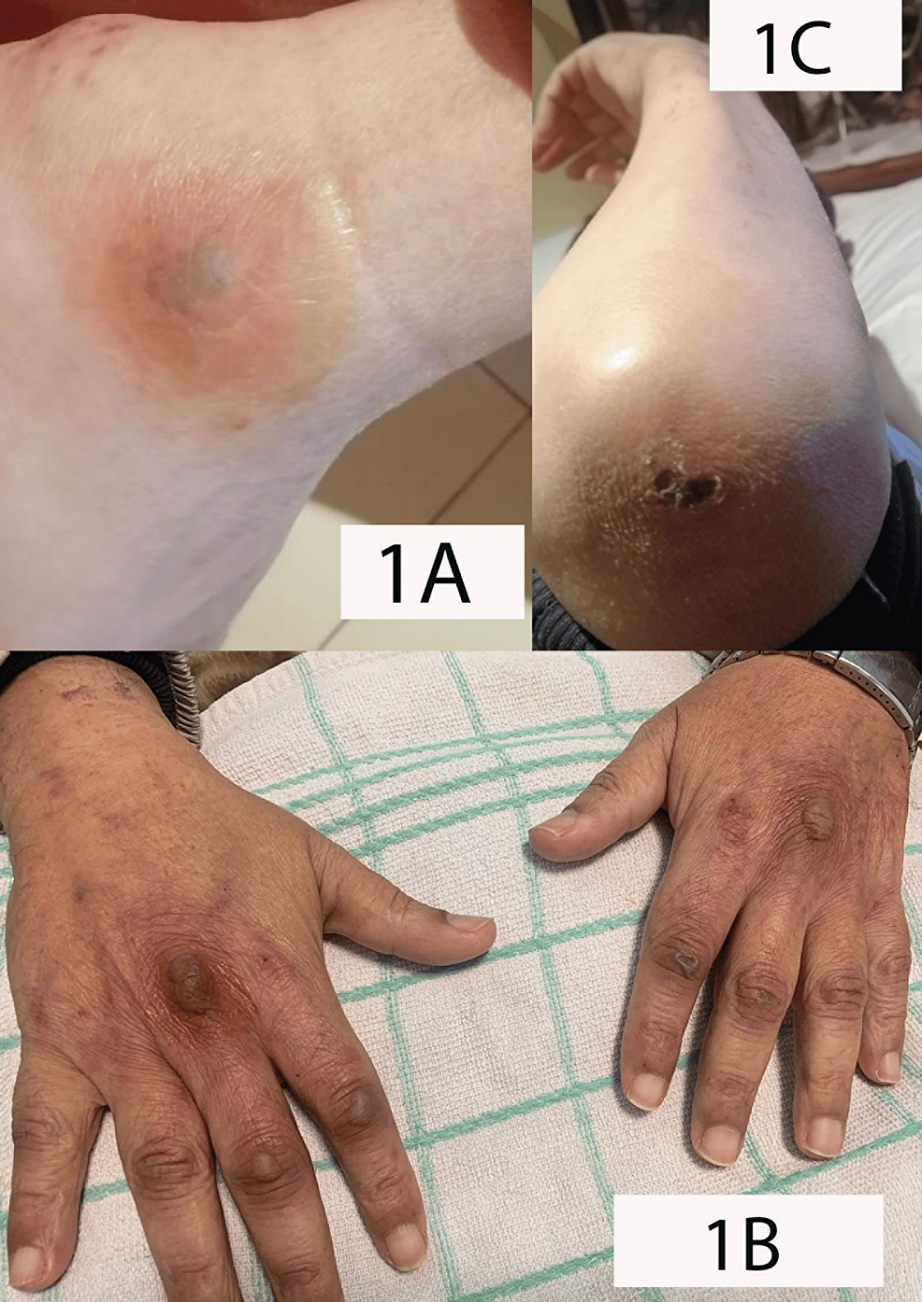
Erythematous papules consistent with Gottron's papules. IA: On the retro-malleolar groove. IB: On the dorsal surface of the metacarpophalangeal and interphalangeal joints. IC: On the dorsal surface of the elbow.

This is a 50-year-old man from northern Morocco who was admitted to the oncology center in Tangier for the management of a locally advanced undifferentiated NPC. He underwent concomitant chemoradiation therapy (CCR) following three courses of induction chemotherapy, which consisted of gemcitabine administered at a dosage of 1 g per square meter of body surface area on days 1 and 8 and cisplatin at a dosage of 80 mg per square meter on day 1, every three weeks. Three months after the treatment ended, he presented total dysphagia, erythematous rash on the extremities, and pelvic muscle weakness making it difficult for him to stand up. The clinical examination revealed a weakness in the paravertebral and proximal pelvic girdle muscles; the patient gets up by supporting himself with his hands on his knees. We also detected a skin rash on the extremities consisting of small erythematous areas spaced by normal skin, along with the formation of papules and macules over some joints, notably the proximal interphalangeal and metacarpophalangeal joints, elbow, and ankles (Figure 1).

**Figure 2:**
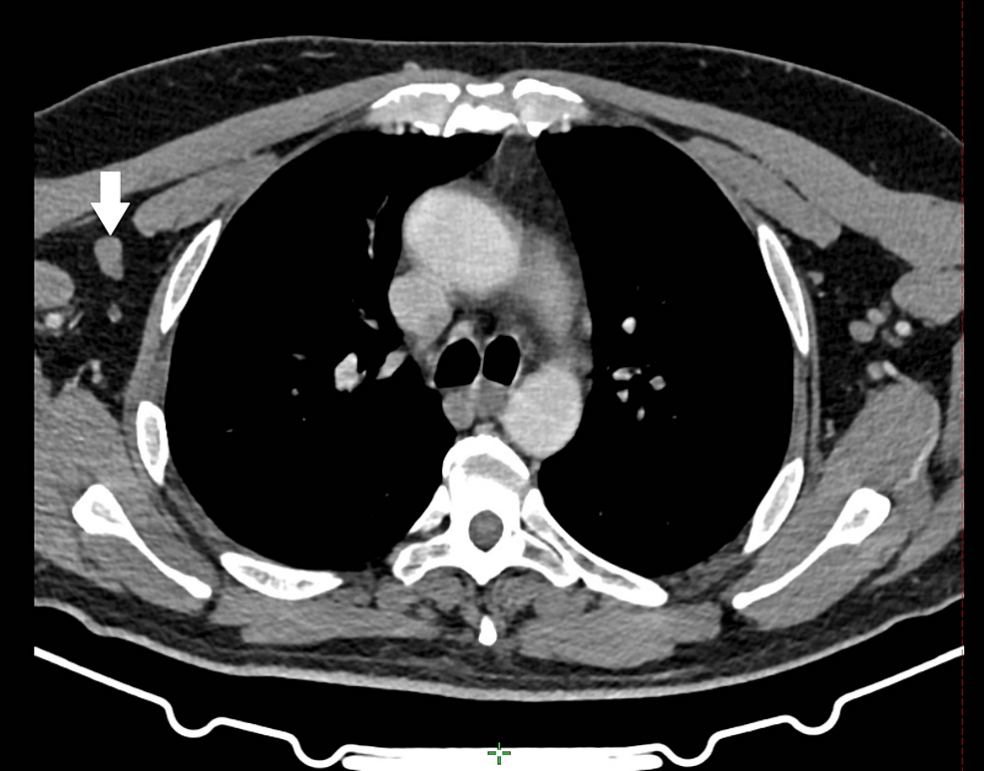
A suspicious right axillary lymph node suggestive of metastatic progression three months after concomitant chemoradiation therapy. White arrow: metastatic axillary lymphadenopathy.

Laboratory investigations showed normocytic normochromic anemia with anisocytosis, thrombocytosis at 705,000 U/l, and an elevated creatine phosphokinase (CPK) at 395 IU/l. After consulting with hematologists, we suspected a paraneoplastic syndrome in view of the patient's history of malignancy and according to the dermatologist's opinion; given the combination of skin abnormalities, increased CPK levels, and muscle weakness, DM was the first diagnosis to be considered. A spinal MRI was requested to rule out spinal cord compression due to vertebral tumor progression, which could explain the onset of neurological deficit; however, no anomalies were seen, and a thoracic-abdominopelvic CT scan showed the appearance of bilateral lung nodules and a suspicious axillary lymph node whose malignancy was confirmed after adenectomy (Figure 2).

**Figure 3:**
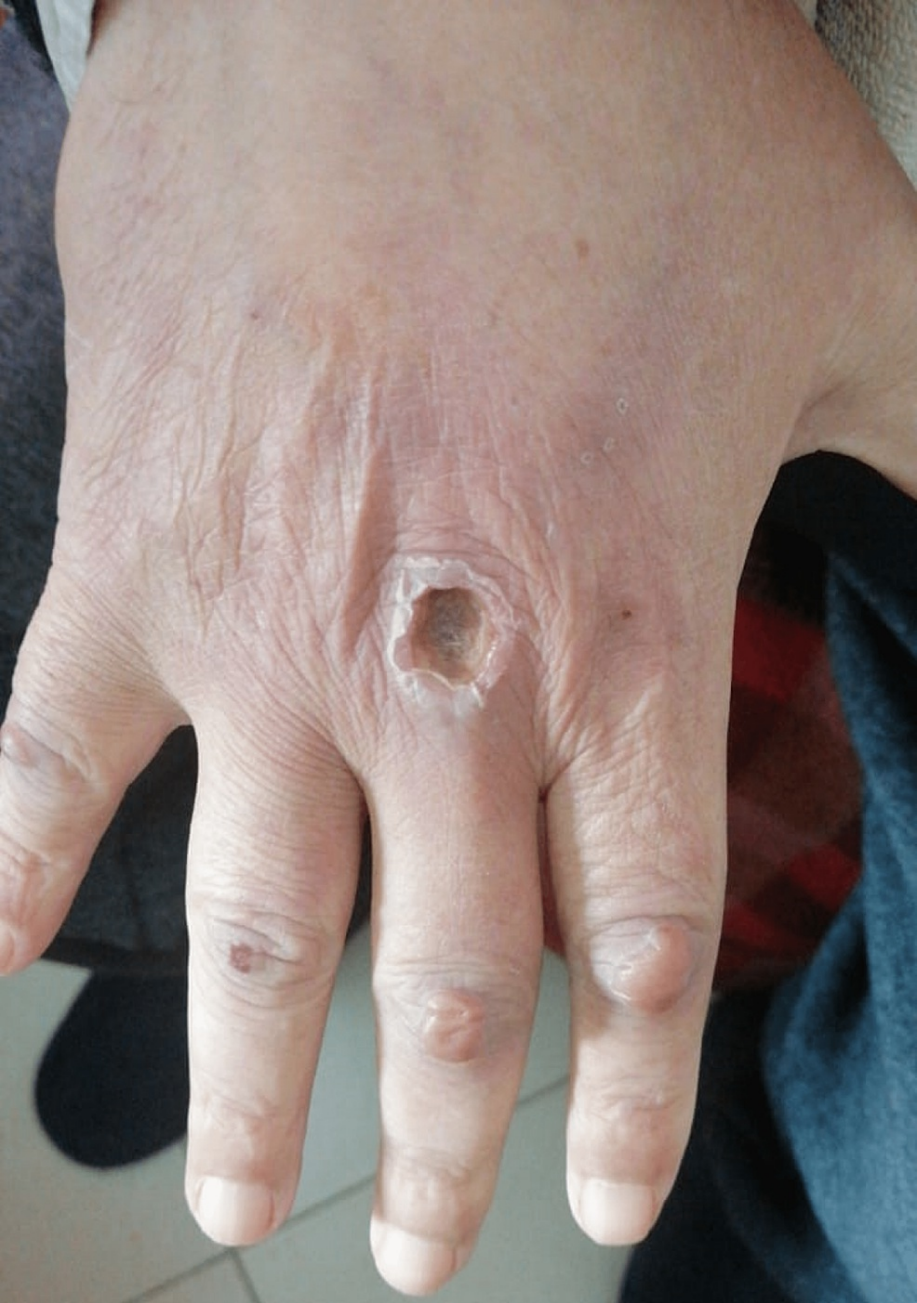
Clinical improvement of Gottron's papules after two weeks of corticosteroid therapy.

Two weeks following the start of treatment, there was a clear clinical improvement of DM after a corticosteroid therapy with a dose of 1 mg/kg of prednisone and a chemotherapy course targeting its metastatic progression, using the same regimen as mentioned above. The patient saw a slight regain of muscle strength, and the skin rash almost disappeared. Regarding the papules, they became smaller, with the appearance of some crusts and flattened blisters, indicating the start of the healing process (Figure 3).

The biological assessment reveals a persistence of anemia but with an improvement in the hemoglobin level compared to the time of diagnosis and normalization of thrombocytes (hemoglobin at 10.1 g/dl and thrombocytes 392,000 U/l).

The patient then received three supplementary courses of chemotherapy with a partial response to the metastatic disease and a complete response to DM. Treatment was stopped in our patient due to poor tolerance and compliance with chemotherapy, and he was monitored. Observation showed disease stability three months following the end of treatment. Actually, he is still clinically, radiologically, and biologically stable after a 15-month follow-up period.

## Discussion

The association of DM with malignancy is infrequent, with several cases of ovarian, lung, and gastrointestinal adenocarcinomas reported. As for NPC, the first case of DM was published in 1969 [5]; since then, several cases have been reported in Asia including Hong Kong, northern China, Singapore, and Malaysia [6,7]. A 6.5-fold increased risk of cancer is associated with DM; this risk is more pronounced in persons over 45 and has been reported primarily in Western and Asian nations [8]. Screening remains necessary in light of this significant risk; however, screening modalities seem to vary from one practitioner to another. 

Given the absence of consensus guidelines in this regard, a conventional cancer screening panel is often used, including tumor markers (CA125, CA19-9, carcinoembryogenic antigen, and prostate-specific antigen) and other tests for women such as mammography, transvaginal ultrasound, and cervical biopsy. Some studies even suggest the use of PET-CT as an effective screening tool with lower direct costs for patients [9]. In North African countries, this association has been reported in Tunisia and Morocco [4,10,11], showing higher incidence between 40 and 50 years and male predominance.

DM appears first in the majority of cases and less frequently at or after diagnosis [6,7,12]. The clinical signs of DM are mainly muscular and cutaneous and may be associated with dysphagia, interstitial pneumonitis, or inflammatory arthritis. Myoparesis is typically proximal and symmetrical, affecting mainly the deltoid and gluteal muscles, as well as the neck flexors [13]. Erythematous papules known as Gottron's papules that appear symmetrically on joint surfaces, especially the metacarpophalangeal and interphalangeal joints, are a common type of cutaneous lesion, either erythema, periungual capillary dilation, or periorbital edema with heliotrope eruption.

According to Bohan and Peter, five criteria [14,15] are used to make the diagnosis: proximal muscle weakness with or without dysphagia and respiratory disorders, myositis confirmed by biopsy of the affected muscle, elevated muscle enzymes, abnormal muscle findings on electromyography (EMG), and a pathognomonic cutaneous eruption, such as Gottron's papules, a rash on sun-exposed areas, periorbital edema, or poikiloderma. At least three of these criteria confirm the diagnosis of DM, and two of them suggest it. The presence of proximal muscle weakness, an elevated CPK, and Gottron's papules supported the diagnosis of DM in our case.

Although the late onset of DM compared to malignancy can be possible [12], the literature suggests that DM frequently occurs around a year before the diagnosis of the tumor [16]. Regarding the tumor stage, the occurrence of DM is typically at the outset, mostly at a localized stage, and rarely at a metastatic stage. According to Duncan and Lau's series, 36% and 3% of patients, respectively, had DM at the metastatic stage of NPC [17]. Due to a relation between the onset of the paraneoplastic syndrome and the elevation of CPK, a first episode or relapse of DM may accompany the relapse or progression of the tumor [16,18]. Immunotherapy, specifically first-line treatments like PD-1/PD-L1 checkpoint antibody inhibitors, is considered among the preferred treatments [19]. However, they are not recommended for patients with auto-immune diseases such as DM. Therefore, chemotherapy regimens like cisplatin-gemcitabine, which are part of first-line therapeutic indications, remain the best therapeutic option to propose for our patient.

Corticosteroids and immunosuppressive therapy are the standard treatment for DM in adults. Prednisone is used at a dose of 1 mg/kg/day without exceeding 80 mg or oral azathioprine (1.5 mg/kg/day) in the first line [3]. We chose corticosteroid therapy for our patient to avoid any added toxicity of immunosuppressive treatment with chemotherapy for his metastatic disease. In a comparative study of 172 patients with NPC, which aimed to evaluate the impact of DM on survival, no difference was demonstrated in local control or five-year progression-free survival [20]. The appearance of DM had no impact on therapeutic response or tumor control in our patient, who currently maintains a very good response to chemotherapy with lesion stability after 15 months.

## Conclusions

NPC with DM is a specific entity that has been increasingly observed recently. Its diagnosis is based on clinical, biological, and pathognomonic histological criteria. The evolution of DM follows that of NPC in most cases and does not seem to affect the overall prognosis. This paraneoplastic syndrome (DM) often precedes or accompanies the diagnosis of NPC. However, it rarely signals metastatic relapse, as in the case of our patient. In conclusion, this association may have a very important role in the screening and monitoring of accompanying neoplastic disease.
